# Automated microfluidic cell culture of stem cell derived dopaminergic neurons

**DOI:** 10.1038/s41598-018-34828-3

**Published:** 2019-02-11

**Authors:** Khalid I. W. Kane, Edinson Lucumi Moreno, Siham Hachi, Moriz Walter, Javier Jarazo, Miguel A. P. Oliveira, Thomas Hankemeier, Paul Vulto, Jens C. Schwamborn, Martin Thoma, Ronan M. T. Fleming

**Affiliations:** 1 Luxembourg Centre for Systems Biomedicine, University of Luxembourg, 7 avenue des Hauts-Fourneaux, L-4362, Esch-sur-Alzette, Luxembourg; 2 Fraunhofer Institute for Manufacturing Engineering and Automation IPA, Stuttgart, Germany; 3 Division of Systems Biomedicine and Pharmacology, Leiden Academic Centre for Drug Research, Leiden University, Einsteinweg 55, 2333CC Leiden, The Netherlands; 4 Mimetas B.V, PO Box 11002, 2301EA Leiden, The Netherlands

## Abstract

Parkinson’s disease is a slowly progressive neurodegenerative disease characterised by dysfunction and death of selectively vulnerable midbrain dopaminergic neurons and the development of human *in vitro* cellular models of the disease is a major challenge in Parkinson’s disease research. We constructed an automated cell culture platform optimised for long-term maintenance and monitoring of different cells in three dimensional microfluidic cell culture devices. The system can be flexibly adapted to various experimental protocols and features time-lapse imaging microscopy for quality control and electrophysiology monitoring to assess cellular activity. Using this system, we continuously monitored the differentiation of Parkinson’s disease patient derived human neuroepithelial stem cells into midbrain specific dopaminergic neurons. Calcium imaging confirmed the electrophysiological activity of differentiated neurons and immunostaining confirmed the efficiency of the differentiation protocol. This system is the first example of an automated Organ-on-a-Chip culture and has the potential to enable a versatile array of *in vitro* experiments for patient-specific disease modelling.

## Introduction

Laboratory automation is becoming increasingly prevalent in the life sciences^1,2^. Automated cell culture has the potential to increase the quantity and the quality of experiments that can be completed in parallel and enables long-term cell culture maintenance with reduced manual labour^3^. Once an automated protocol is established, a robot can operate continuously without fatigue and with the same consistency and accuracy^2^. Likewise, once established an automated imaging system can take repeated measurements over a long period without intervention^4^. The combination of robotic cell culture and automated imaging has a wide range of biological applications. A leading example is their use to distinguish causation from correlation in the pathogenesis of neurodegenerative diseases by longitudinal measurement of human *in vitro* disease models^5^. Laboratory automation requires precise specification of, and enables fine control over, many experimental protocol parameters, such as dispensing speed, cell culture conditions, fluid temperature and measurements. This enhances experimental reproducibility by reducing variance between replicates^6^. *In vitro* cell culture automation facilitates faithful replication of certain *in vivo* physiological conditions as it enables quantitative control over key experimental parameters, e.g., perfusion rate^7^. This increases the validity of employing an *in vitro* model to represent an *in vivo* system, in health or disease, thereby accelerating biomedical research.

During manual cell culture, procedures involving liquid handling, such as dispensing media, aspiring media, and movement of liquid samples between containers, are essential to all protocols. Therefore, when a cell culture protocol is automated, a liquid-handler and a robot for transposition of receptacles, are two of the most important devices. There are two types of technologies used in liquid-handler: *contact* and *non-contact dispensing*^2^. On one hand, to dispense a precise volume, contact dispensing requires the head of the tip holding the fluid to touch the bottom of the substrate; for instance, the bottom of a well or to touch the liquid surface. On the other hand, non-contact dispensing does not require any contact between the tip and the substrate or liquid surface for liquid release. Dispensing can require the handling of very small volumes, as low as a few nano-litres, so the technological advances in liquid handlers have focused more on dispensing than on aspiration^2^. Low volume dispensing and aspiration are especially required for microfluidic cell culture^7^. The robot to move receptacles can be a robotic arm or a gantry robot with a gripper for receptacles^2^. A gantry robot only moves in Cartesian coordinates, where the three principal axes of control have linear actuators.

The choice of devices used in laboratory automation should be based on their intended uses, flexibility, purchase costs and maintenance costs. Selecting the components of an automated plant usually entails having to purchase devices from different manufacturers, as no single firm supplies all of the devices that might be required to automate a laboratory protocol. Therefore, all of the components must be amenable to software integration in order to be able to function as a single autonomous plant. *Computer scripting* achieves integration by assigning a master software that communicates directly with all devices^8^. In this approach, assuming that all the devices are able to send and receive commands, a communication protocol must be implemented that is compatible with each individual device. However, this approach requires the master device software to recognise every other device using an idiosyncratic communication protocol. This approach can be very expensive and challenging to implement. Alternatively, *Standardisation in Laboratory Automation* (SiLA, http://www.sila-standard.org/) is a consistent and efficiently extensible approach for integration of laboratory automation devices, based on a standard protocol specification for exchanging structured information in a client-server model of communication. Furthermore, SiLA defines over 30 standard device classes used in the field of life sciences, including incubators, microscopes, de-lidders and liquid handlers^9^. For each device class, a list of required and optional functions are proposed to standardise the software communication within a laboratory automation plant. This approach standardises the communication between all of the devices of a plant, regardless of the manufacturer, and a SiLA compatible process management software can then be used to control each SiLA compatible device, without any modification.

Parkinson’s disease is characterised by cell death in selectively vulnerable parts of the nervous system^10,11^. These neuronal losses include cholinergic neurons, noradrenergic neurons and dopaminergic neurons which play a critical role in brain function by releasing a neurotransmitter called dopamine^12–16^. The loss of dopaminergic neurons is the main reason behind the motor symptoms of Parkinson’s disease patients^17^. The study of Parkinson’s disease at the cellular level has been facilitated by the use of *induced pluripotent stem cells* (iPSCs) technology^18^. iPSCs are embryonic-like stem cells that have been derived from somatic cells, skin fibroblast, via reprogramming^19^. Reinhardt *et al*.^20^ developed a protocol to generate human neuroepithelial stem cells (hNESCs) from iPSCs. These hNESCs can in turn be differentiated into many neuronal cell types, including midbrain-specific dopaminergic neurons, critical to the *in vitro* modelling of Parkinson’s disease pathogenesis.

Microfluidic cell culture concerns the design and implementation of devices and protocols for the culture, maintenance and perturbation of cells in micro-scale fluid volumes. The reasons behind the popularity of microfluidic cell culture are both economic and scientific. Cell culture reagents are expensive, and the amounts used in microfluidic cell cultures are much less than in macroscopic cell culture^21,22^. Microfluidic cell culture also has the potential to lower the ratio of extracellular to intracellular fluid volumes, thereby decreasing the temporal lag in extracellular response to molecules transported across cell membranes, e.g., in exometabolomic analyses^23–25^. With the advent of Organ-on-a-Chip technology^26^, microfluidic cell culture has developed tremendously and includes examples of perfusion culture, co-culture and three dimensional cell cultures^27–29^. Moreover, miniaturisation enables multiple experimental replicates within a geometrically confined experimental footprint. Thus far, no examples are known of an Organ-on-a-Chip operation in an automated setting, although few hold the promise to do so^30^. Even though the combination of automation, microfluidics and cell culture technologies allows the screening of multiple environmental conditions in parallel^31,32^, as well as enabling regular live cell culture monitoring^33,34^ at a temporal resolution impractically in a manual setting. Therefore, laboratory automation technology is key to unleash the full potential of microfluidic cell culture. We previously developed a microfluidic titer plate for three dimensional microfluidic cell culture, called an OrganoPlate^28^. Subsequently, we implemented the differentiation of hNESCs into three dimensional networks of electrophysiologically active dopaminergic neurons into the OrganoPlate^35^. The microfluidic titer plate was designed for compatibility with laboratory automation, but this has yet to be exploited. The manual culture of human pluripotent stem cell derived cells within the microfluidic titer plate has also been established, but the potential for automation has also not yet been exploited.

Herein, we report the integration of developmental biology, microfluidic cell culture and laboratory automation technology to generate a flexible automated, enclosed microfluidic and macroscopic cell culture observatory, termed the *Pelican*. We elaborate on each device in the Pelican, as well as the SiLA software integration approach used to realise an automated system. We illustrate the functionality of the Pelican for automated cell culture and differentiation of human neuroepithelial stem cells into dopaminergic neurons, within a three-dimensional microfluidic device^28^. We monitored the health of the cells throughout the experiment with an automated image acquisition pipeline. After 24 days in culture, we assessed the outcome by characterising known features of dopaminergic neurons by calcium imaging and immunofluorescence assays. Three dimensional imaging revealed mature and interconnected neuronal populations within microfluidic cell culture chips. The Pelican is a modular automation system, compatible with implementation of a variety of automation platforms, where cost-effective flexibility is maximised to allow for replacement or further expansion of platforms by integration of new devices. Microfluidic cell culture has already been manually integrated with iPSC technology^35^. Our work integrates an automated system with an Organ-on-a-Chip stem cell culture.

## Results

### System Construction and Design

**Figure 1:**
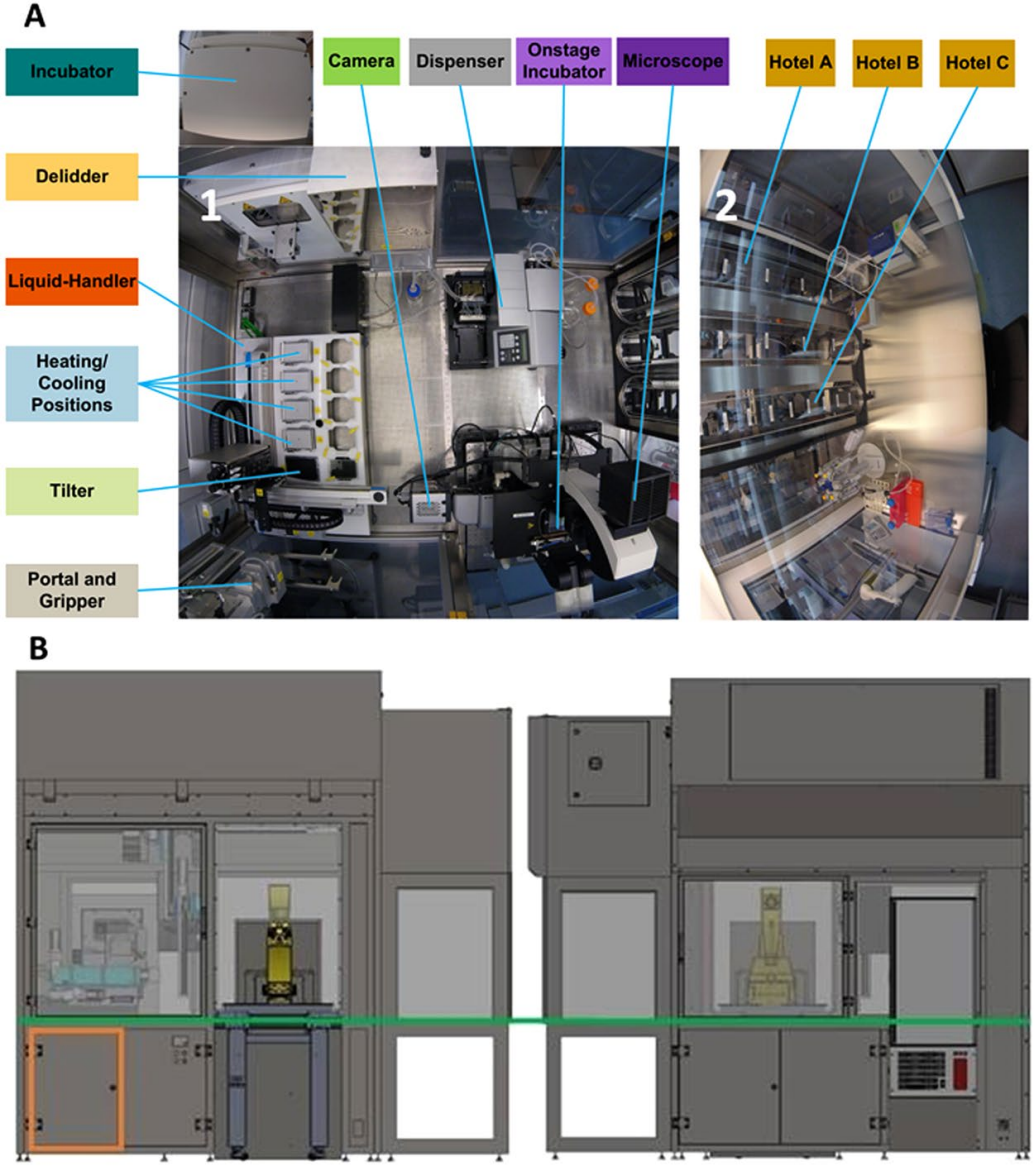
Pelican automated cell culture observatory. (**A**) Top view inside the Pelican automation workstation without housing: (1) Wide angle lens image of the automated enclosure. (2) Wide angle lens image of the adjacent manual cell culture bench. (**B**) Outside view of automated culture system (top). Front (bottom left) and rear (bottom right) views of Pelican with housing. Yellow (imaging station), light blue (liquid handling station), green (level of stainless steel work surface) and orange (waste containers). The colour codes of the devices labels in (**A**) and Fig. S1 match.

Figure 1 illustrates the automated cell culture enclosure (see Supplementary Figs S1 and S2), constructed and assembled according to the hardware design and the plant control architecture. The different devices and their use in the automated system are detailed in the Supplementary Experimental Procedures.

#### Hardware selection

In brief, in the Pelican, a four-axis (X-Y-Z-_θ_) gantry robot is used in combination with a gripper, that allows many types of substrates to be handled and services all devices with substrate from above. The robot was attached to the top of the stainless steel frame support inside the housing through rails that allow the movement of the robotic arm along three axes. This maximised the modular capacity of the Pelican because the rails determine the robotically *useful space* of the system, in contrast to other automated systems with a fixed rotating robot arm, which often has limited reach. The useful space is the space that is available to potentially hold new devices, adding to the functions of the automated platform.

In the Pelican, a ZEUS pipetting module (Hamilton Inc.) with disposable tips combines the precision of contact dispensing with the versatility of non-contact dispensing. In addition, a liquid dispenser with only non-contact dispensing was also implemented in the system. The liquid dispenser is less precise than the liquid-handler. However, it is much faster as it can handle up to 96 wells per step compared to 4 wells per step for the pipetting modules. Despite its shortcomings, the contact dispensing function of the ZEUS is especially useful for microfluidic cell culture where very small volumes must be dispensed. In addition, the contact dispensing helps to make sure that the dispensed media is bubble free. This is very important as with low flow rate non-contact dispensing, bubbles that arise can imped the flow of fresh media, which could ultimately starve the cells in a chip. Contact dispensing is very precise for dispensing small volumes. However, this precision is dependent on dispensing at an exact location, which is not always possible as the dispensing tip cannot always physically access the well to make contact with the liquid^1^.

With contact dispensing, well cross-contamination is a risk as there is direct contact between the tip and the liquid in the destination well. Therefore, a cleaning protocol was implemented after each dispensing step. This does not promote speed and high-throughput capabilities so sought after in laboratory automation, however, the contact dispensing was only utilised for the initial loading of media to avoid the introduction of bubbles in the dry medium lane. Non-contact dispensing does not require any contact between the dispensing tip and the liquid. This helps to avoid cross-contamination, and promotes the integrity of the well. Non-contact dispensing is very popular in laboratory automation because it is versatile, and it is easy to dispense to any area of a well regardless of geometry such as undercuts, so long as there is an opening on the well^36^. As a result, the non-contact dispensing methodology was utilised for all subsequent media changes.

#### Software integration

Each of the devices of the Pelican are physically connected via Ethernet to a computer (Precision T7600, Dell Sa, Mamer, Luxembourg), according to the plant architecture illustrated in Fig S1. The SiLA standard was consistently implemented for networking and integrating the devices and components. A driver development kit (DDK, Fraunhofer IPA, http://www.sila-standard.org/driver-information-platform/fraunhofer-ipa/sila-driverdevelopment-kit/) enabled the development of SiLA compliant drivers for each of the devices, if it was not available from the manufacturer. Incorporating a driver and converting the commands occurred in Laboratory Automation Control Suite (LACS, Fraunhofer IPA).

In brief, LACS is a programming environment for laboratory automation. It incorporates three software packages: LacsDriverCore, LACS graphical user interface (GUI) and LACS Config Editor. LacsDriverCore reads the SiLA drivers of the devices generated by the DDK, hence, it connects the devices to a computer. The LACS GUI is the process management software, the interface between a device and an operator (via the computer) through a loaded SiLA driver. The running of all existing protocols through LACS GUI merely requires the identification of the required protocol and the input of the required plate name by the operator. LACS Config Editor is used to draft automated protocols. LACS does not make any process-specific decisions, nor does it analyse any data. In the occurrence of an unexpected event (a device failure or any other error), the operator is always prompted to assess and rectify the error or the event.

The safety features around the housing, the hotels and the incubator of the Pelican (sensors to read the states of the components), and the operations of the de-lidder and the stage incubator were all controlled through a digital and analogue logic module (UR20-FBC-MOD 1334930000, Weidmuller GmbH & Co. KG, Germany). A digital and analogue logic device has a binary set; a binary input and binary output. It is usually used for a maintenance device or to control a device with a simple binary command such as for a valve ON/OFF and a light switch. A single weidmuller SiLA driver was installed to control all devices connected to one logic module.

Software (Labware Manager, Fraunhofer IPA) handles all positions inside the Pelican plant and all substrates. In the first case it holds the information, which position is occupied and with which substrate and which free position could potentially hold which type of substrate. The software knows each position and status as well as each substrate in the plant including position and type. All data are stored in an open source PostgreSQL database (PostgreSQL, https://www.postgresql.org/). Like the hardware devices, this virtual device has a SiLA communication interface and a Windows 7 graphical user interface to view the stored data.

The integration of new devices requires two or three steps depending on the complexity of the device and the availability of compatible drivers. The first step requires the generation of a driver for the new devices. If a SiLA driver is already available through the device manufacturer, open source, or if a commercial software that can be referenced in Microsoft Visual basic is available, then one can directly use the existing driver and move to the second step. Otherwise, one has to use the Driver Development Kit to generate a SiLA compatible driver. The DDK already possesses the framework to easily write the driver for most laboratory devices such as a camera, a microscope or a liquid dispenser. One needs only to add the commands provided by the manufacturer of the device. The second step is completed by assigning the new device a SiLA IP/Port. Once the SiLA driver of the new device is written and tested, the final step is to integrate it into the Pelican. This is done, by opening the driver in LACS Configuration Editor and saving the device in the list of working devices in the Pelican.

### Microfluidic device

**Figure 2:**
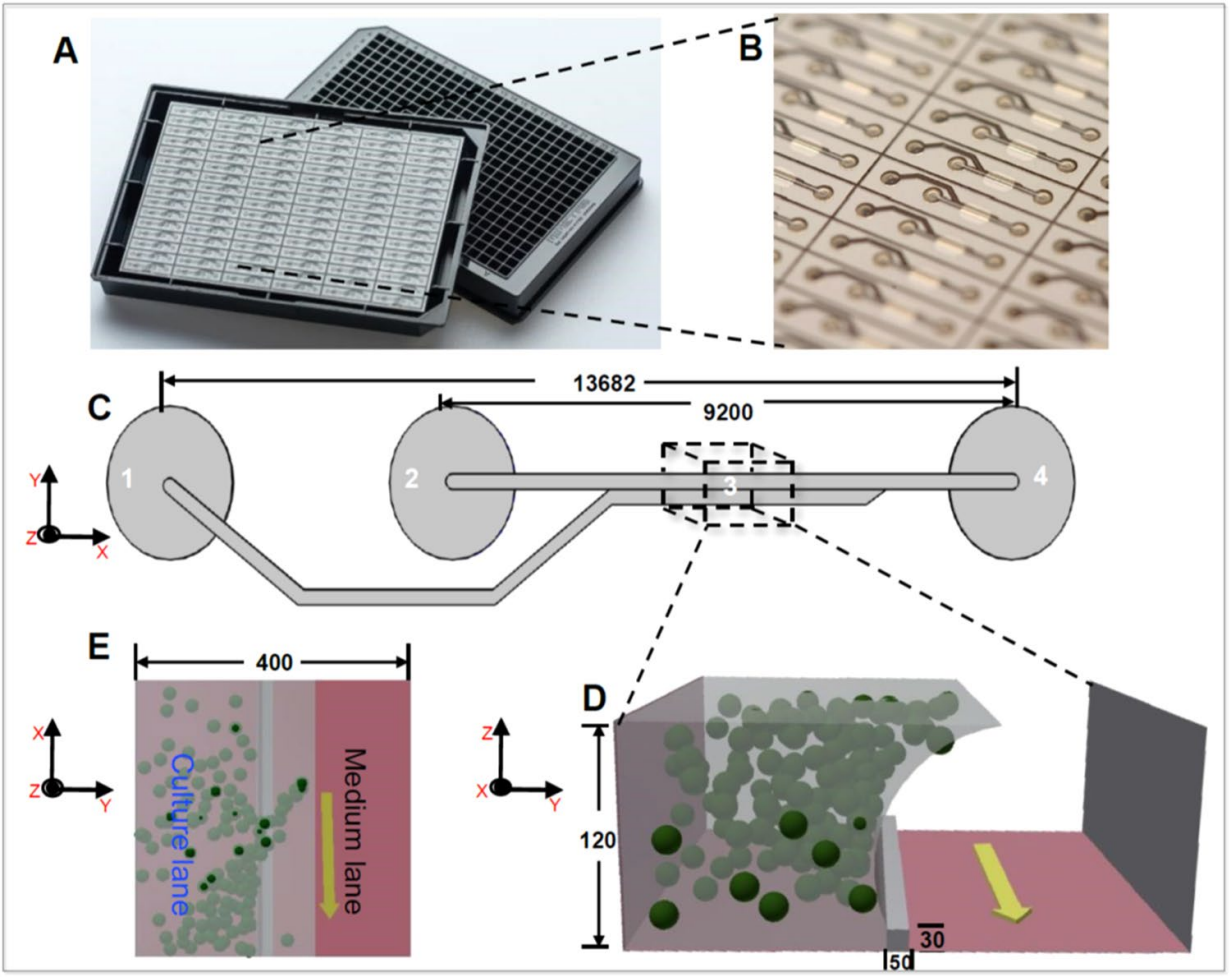
Microfluidic cell culture device: OrganoPlate. (**A**) Photograph of underside (left) and upper views (right) of an OrganoPlate. (**B**) bottom plate of OrganoPlate with selective chips. (**C**) A schematic of a single 2-lane chip; 1 = Gel inlet, 2 = Medium inlet, 3 = Observation window, 4 = Medium outlet. (**D**) A transverse section of a culture chamber showing the direction of the flow in the medium lane (yellow arrow), the phaseguide and the culture lane with suspended spheres representing cells embedded in Matrigel. (**E**) Top view of transverse section of a culture chamber with phaseguide, medium and culture lanes. All dimensions in μm.

A 2-lane *OrganoPlate* (#*9603-200B*, Mimetas BV, Leiden, The Netherlands) consists of a stratified array of 96 microfluidic chips embedded in a customised 384-well microtiter plate format^28^ (Fig. 2). Each chip consists of a single microfluidic chip contained between two pieces of glass: a top plate with holes corresponding to the underside of selected wells, and a bottom plate. Each chip is connected through 4 neighbouring wells and 2 lanes: one *gel inlet well* for loading of gel-embedded cells into the culture lane, one *medium inlet well* connected to one *medium outlet well* through a medium lane. The flow of media is driven by a pressure drop between the aforementioned 2 wells. The fourth well is used as an *observation window* for monitoring the quality of cells through an inverted microscope. The culture and medium lanes are separated by a *phaseguide*, preventing the gel-embedded cells from flowing into the medium lane. A phaseguide is a patterned pinning barrier that controls the liquid-air interface by forcing it to align with the ridge, hence, guiding the fluid flow into the appropriate lane^27^.

### Automating the differentiation of human neuroepithelial stem cell into dopaminergic neurons

In order to biologically validate the automated system, and the ability of the Pelican to differentiate cells in microfluidic device, the assay developed by Reinhardt *et al*.^20^ and adapted into the OrganoPlate by Lucumi *et al*.^35^ was used to develop an automated pipeline to differentiate hNESCs into three dimensional networks of electrophysiologically active dopaminergic neurons in the OrganoPlate. The first step of the assay involve loading 0.8 μl of matrigel/hNESCs mixture into the culture lane.

**Figure 3:**
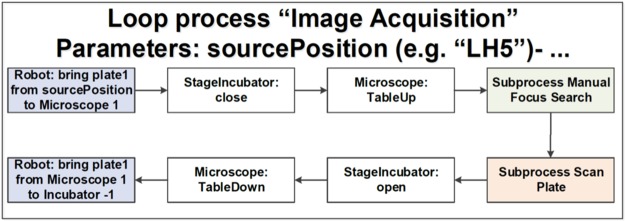
Semi-automated image acquisition pipeline. See Supplementary Fig. S5 for more details.

**Figure 4:**
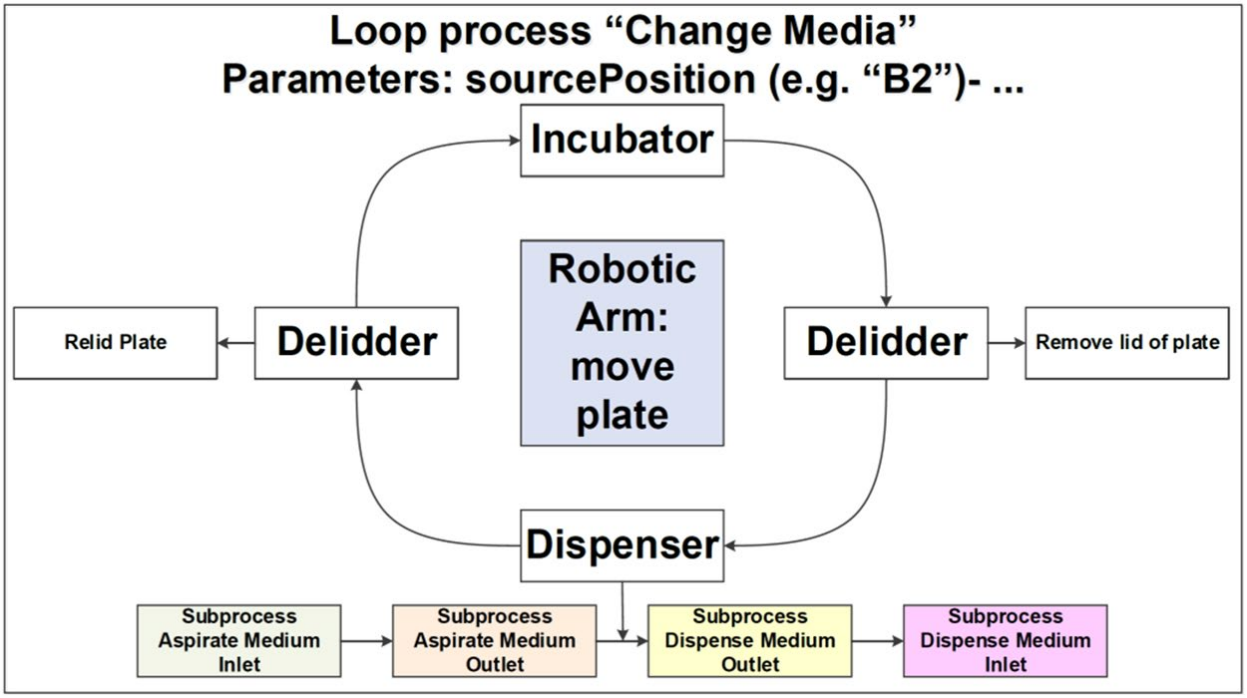
Automated media change pipeline. See Supplementary Fig. S4 for more details.

The day after loading the gel-embedded hNESCs into the culture lane of the OrganoPlate, a *semi-automated image acquisition protocol* was executed through LACS to qualitatively assess the health of the cell culture. An appropriate field of view was selected manually and focussed. The subsequent automated image acquisition protocol (Fig. 3) consisted of first setting the environmental condition of the onstage incubator to 5% CO_2_ and to a temperature of 37 °C. Second, the robotic arm moved the plate from the storage incubator to the microscope. Third, the microscope scanned through all the observation windows of the OrganoPlate, and the camera took an image of each observation window. Fourth, the plate was transported back to the incubator by the robotic arm. Immediately after the automated image acquisition, the *automated differentiation of hNESC into dopaminergic neurons protocol* (Fig. 4) was executed through LACS. This protocol consisted of first instructing the robotic arm to move the plate from the storage incubator to the de-lidder to remove the lid from the plate, before moving it to the liquid dispenser. Second, the dispenser aspirated media from each medium inlet and outlet well. Third, the dispenser replenished the media in each medium inlet and outlet well respectively. Fourth, the robotic arm moved the plate to the de-lidder to put the lid back before placing the plate inside the storage incubator (Supplementary Video 1). These two protocols were run in this order every two days for 24 days.

### Human neuroepithelial stem cell culture differentiation

**Figure 5:**
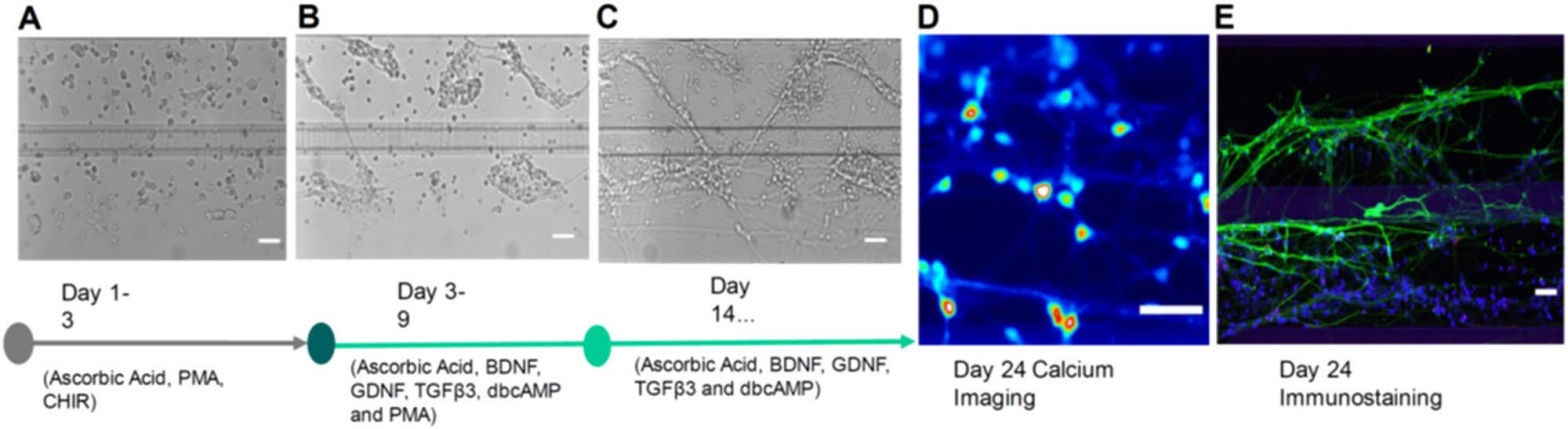
Dopaminergic neuronal differentiation. Bright field images of hNESCs with media components at (**A**) 1 day (**B**) 4 days and (**C**) 21 days after seeding. (**D**) Calcium imaging frame of a firing event taken at day 24 after seeding. (**E**) Immunofluorescence image illustrating the neuronal composition inside a culture chamber. All scale bars are 50 μm. Legend as in Fig. 6.

We utilised the Pelican and the existing protocol described by Reinhardt *et al*. and Lucumi *et al*.^20,35^ to fully automate the differentiation of hNESC into dopaminergic neurons inside a stratified three-dimensional microfluidic device. After manually seeding hNESC in an OrganoPlate, a microfluidic cell culture device with 3D capabilities compatible with laboratory automation, the hNESC were distributed in three dimensions within the culture lane, but also adjacent to the meniscus (Fig. 5A). Thereafter the Pelican executed an automated differentiation protocol to start and maintain the differentiation process. Initially. this required a liquid dispenser to aspirate the maintenance medium and dispense the differentiation medium with PMA, via a dispensing cassette, into inlet and outlet wells of the OrganoPlate. Thereafter, it required incubation and regular media replenishment.

**Figure 6:**
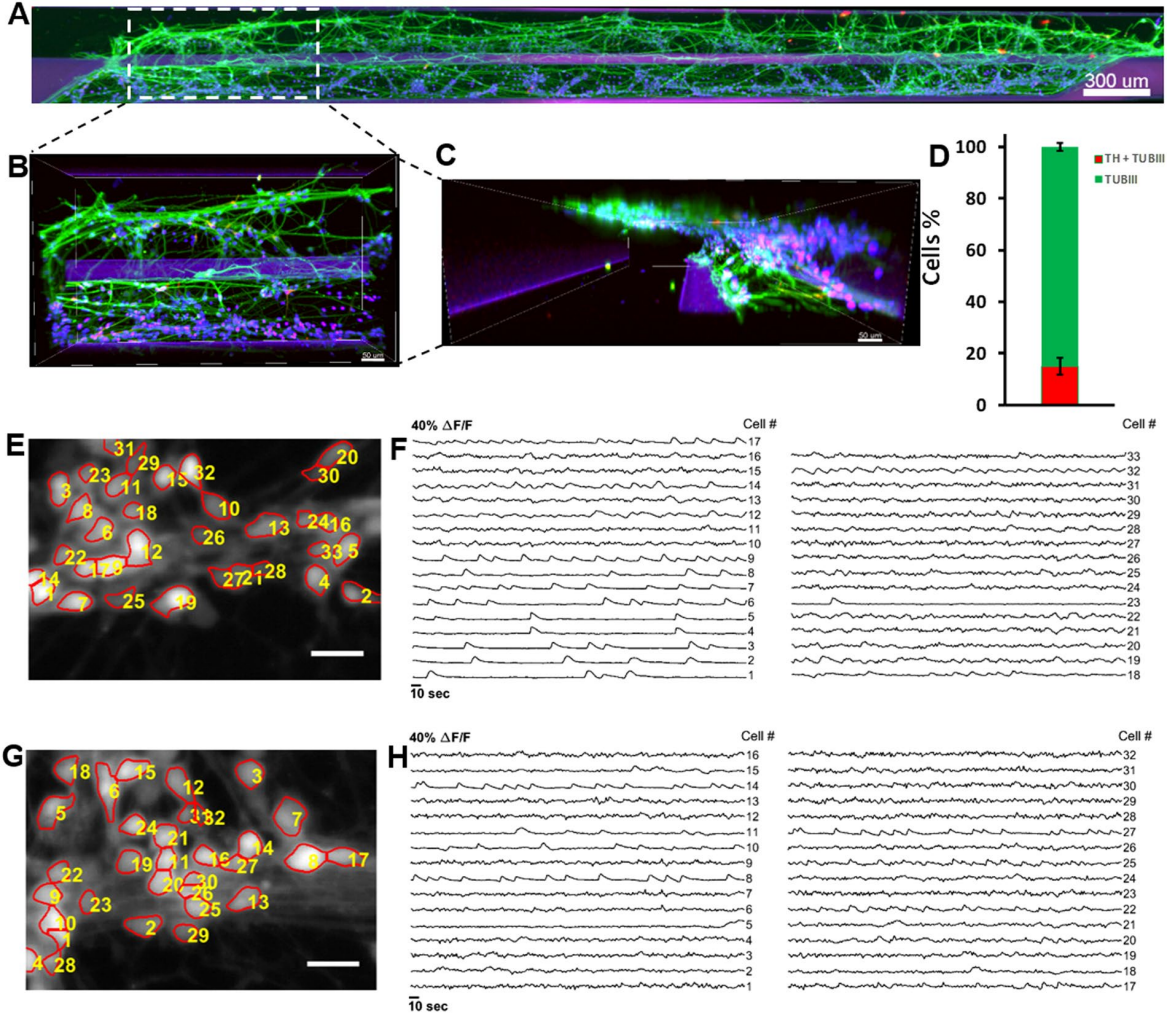
Immunostaining and automated cell segmentation of calcium time-series hNESC differentiated in chips of the OrganoPlate inside the Pelican. (**A**) Top view of an entire chip in the OrganoPlate showing differentiated wild type neurons (K7 cell line) immunostained for nuclei with Hoechst (blue), TUBβIII (green) and tyrosine hydroxylase (red); scale bar 300μm. (**B**) Enlarge top (**B**) and front (**C**) views of selected area; scale bar 50μm. (**D**) Differentiation efficiency of neurons positive for TUBβIII and tyrosine hydroxylase. (**E**) Mean fluorescence frame of a calcium time-series of WT population with segmented regions of interest corresponding to individual neurons and (**F**) their corresponding fluorescence traces. (**G**) Mean fluorescence frame of a calcium time-series of PINK1 mutants with segmented regions of interest corresponding to individual neurons and their corresponding fluorescence traces (**H**).

During the differentiation, cells started to form aggregates, and morphological changes started to appear, such as acquisition of cellular polarity and projection of processes representative of neuronal morphology (Fig. 5B). An automated protocol replaced the medium with PMA with medium without PMA, and differentiated neurons started a maturation process accompanied by acquisition of a more evident neuronal morphology (Fig. 5C). Differentiated neurons projecting their processes can be observed in the culture lane and in the area occupied by the meniscus, located in part of the medium lane. Neuronal activity of differentiated cells was tested using calcium imaging (Fig. 5D) and morphological and phenotypic characteristics like neuronal processes positive for TUBβIII (green) and the amount of tyrosine hydroxylase positive neurons (red) were characterised using an immunostaining assay (Fig. 5E). A top view of the culture chamber of a chip with hNESC (Fig. 6A), shows that cells were distributed in the entire culture chamber, Hoechst nuclear staining (blue). Furthermore, their processes have been projected in all directions of the culture lane, as well as on the meniscus part in the medium lane. It can also be seen that the neuronal networks and the level of connectivity of the differentiated neurons is homogeneous in the entire culture chamber, denoting an even effect of the differentiation protocol, as well as the efficiency of perfusion in the medium lane (Fig. 6A).

Figure 6B enlarges the view of a specific area of one chip with differentiated neurons from hNESC. Neurons positive for TUBβIII (green) and positive for tyrosine hydroxylase (red) are located in both culture and medium lanes. However, all cells appear to migrate towards the medium lane. This is confirmed in the front view of the enlarged area (Fig. 6C), where the extent of the area occupied by the meniscus in the medium lane is clear, in addition to the degree of cells located on the meniscus on the medium lane. On average, the efficiency of differentiation for tyrosine hydroxylase positive neurons in 3 chips of the OrganoPlate with hNESC differentiated neurons was 15% of all neurons (Fig. 6D), which is in accordance with values reported previously in analogous manual, microfluidic and macroscopic cell culture systems^20,35^.

After 24 days, we were able to obtain midbrain-like, mature dopaminergic neurons in 96 three-dimensional self-contained microfluidic chips. Regular pictures were taken during differentiation for quality control and morphological study (Fig. 5A–C), and end point assays are represented in Fig. 5D,E to illustrate the fate of the differentiation.

### Calcium imaging and immunostaining assays

To probe the neuronal activity of cells cultured in an OrganoPlate within the Pelican, we used Fluo-4-based calcium imaging. We acquired time-series of representative culture chambers of WT and PINK1 p.I368N-mutated populations at day 24 of differentiation (Supplementary Video 2). Analysis of those calcium imaging data revealed spontaneous neuronal activity in differentiated control and PINK1-mutant neurons in an OrganoPlate, within the Pelican. We detected individual cells by applying an automated cell segmentation algorithm^37^ to the raw calcium imaging data. Figure 6E,G illustrate segmented mean fluorescence frames of representative culture chambers of an OrganoPlate with WT and PINK1-mutated neurons respectively. Fluorescence traces were then measured for each segmented cell to assess their activity (Fig. 6F,H). Some of the fluorescence traces reveal calcium transients indicating neuronal firing events (Fig. 6F e.g. signal #2, 3, 9 and Fig. 6H e.g. signal #8, 14, 27). Fluorescence traces revealed different firing patterns of the differentiated neurons. Some of these traces exhibited regular firing patterns, as opposed to other ones, corresponding probably to dopaminergic neurons, similar to what has been previously reported by Lucumi *et al*.^35^ in adjacent manual culture.

## Discussion

We assembled an automated cell culture observatory, termed the Pelican. It was optimised for the long-term cell culture maintenance of neurons inside three-dimensional microfluidic devices. The implementation followed a modular automation design, with generous space for further devices, and is flexible in terms of the automation platforms that can be implemented. For example, the system allows the automation of seeding, feeding and other cell culture processes that could initiate and maintain cell lines in any standard microtiter plate format. In this regard, we carefully selected the robotic system, the pipetting technologies and the techniques to integrate all the components of the system into a single plant.

Many methods have been developed over the years to address the issue of standardisation and easy integration of new devices into existing laboratory automated plants^8,9,38–41^. In the Pelican, Standardisation in Laboratory Automation (SiLA) was chosen for the integration of the devices. SiLA standardises the interfacing, integration and data representation in a simple single XML schema for all devices^9,42^. SiLA standardises the communication between process management software and one or more devices. SiLA defines common commands per *device class* and the common *device states*. SiLA defines the device classes to achieve these functionalities such as an incubator and a liquid-handler. Each device class will have the required commands for the core functionalities as well as *optional commands* for extended functionalities that are not necessarily present in every device of the same class. In this regard, a SiLA standard common command dictionary was developed for every single device class where the commands such as setParameter and getParameter and the expected return are known. This allows a process management software to automatically generate the required commands for every device class. Each component of the Pelican was chosen based on cell culture needs without taking into account the manufacturer of each device. The integration of any new device would require sufficient space within the useable area and possibly the development of a SiLA driver (tutorial in user manual available upon request). The modular hardware and software integration flexibility is a key advantage of the Pelican design compared to other automated cell culture systems^43–48^.

In order to demonstrate the biological utility of the Pelican, control and PINK1-mutant human neuroepithelial stem cell lines were automatically differentiated into midbrain specific dopaminergic neurons. Calcium imaging of spontaneously firing neurons, as well as immunostaining for neuronal markers demonstrate that, neuroepithelial stem cells could be successfully maintained and were spontaneously active within the OrganoPlate inside the Pelican. Further analysis of fluorescence traces for additional cell lines with different genetic backgrounds would be necessary to quantify any difference in phenotypic characteristics of neuronal activity between control and PINK1 mutant neurons. The differentiated neurons can be maintained inside the OrganoPlate for at least 100 days. The Pelican demonstrates proof-of-concept for automated generation of personalised *in vitro* neuronal models from human neuroepithelial stem cells via a microfluidic cell culture approach.

The Pelican is designed for longitudinal analysis of many personalised cellular models exposed to a few perturbations, rather than single, end-point analysis of one cellular model exposed to a large number of perturbations, as for instance is the focus in high throughput drug screening. Therefore, we envisage that such automated system be applied to stratification of patients with complex diseases. In addition, the Pelican can be used of automated cell culture to enable comprehensive phenotyping of large, parallel sets of personalised, *in vitro*, midbrain-specific, dopaminergic neuronal models of Parkinson’s disease. By integrating the data generated with a generic mechanistic computational model of the underlying biochemical network of a dopaminergic neuron, each personalised computational model then becomes a coherent representation of our information about the cell autonomous characteristics of Parkinson’s disease in each patient. Such personalised computational models, and thereby the corresponding patients, are then amenable to stratification with a range of powerful stratification tools. Stratification based on personalised computational models of data is statistically superior to stratification based on the personalised data alone, as the former explicitly incorporates the wealth of prior biochemical information known about midbrain dopaminergic neurons. Ultimately, this will accelerate the translation of basic biomedical knowledge from the laboratory to the therapies with clinical impact.

Increasing demands for reproducibility, parallelisation and longitudinal observations are driving cell culture research toward automation. We developed a novel automated cell culture observatory that enables long-term maintenance and longitudinal optical measurement of cellular parameters in Organ-on-a-Chip platforms. We demonstrate the use of this platform to successfully automate the generation of personalised *in vitro* neuronal models from human neuroepithelial stem cells. We demonstrate the feasibility of semi-automated image acquisition on this platform and compatibility with different real-time and end-point assays. It is the first time that an Organ-on-a-Chip platform is applied in an automated setting. It holds great promise for patient stratification by enabling comprehensive phenotyping of large, parallel sets of personalised, *in vitro*, models of complex diseases.

## Methods

### System Construction and Design

**Figure 7:**
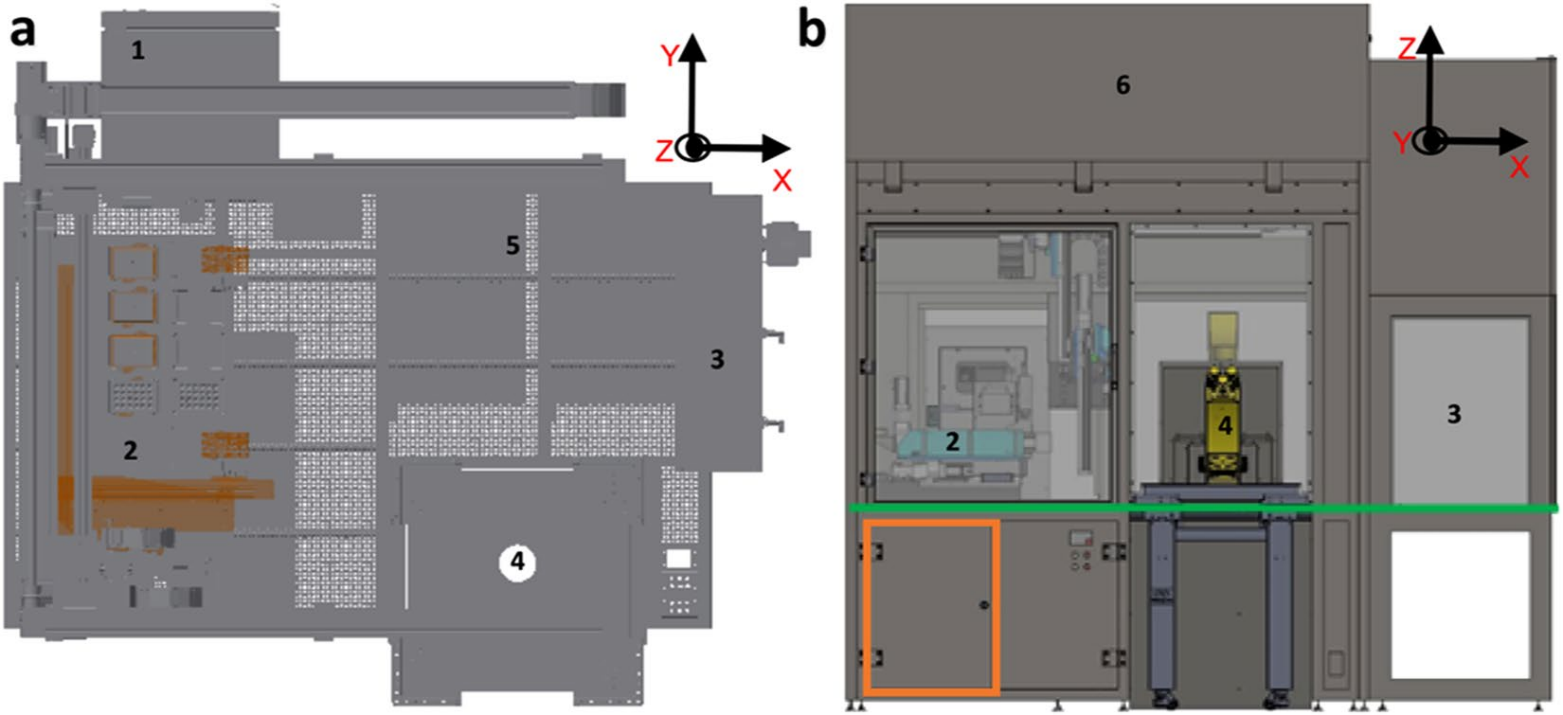
CAD drawing of automated workstation. (**a**) Top view inside the Pelican without housing. (**b**) Front view of the Pelican with housing. Yellow (imaging station), light blue (liquid handling station), green (level of stainless steel work surface) and orange (waste containers). Perforated surface = work surface. (1) Storage incubator. (2) Liquid-handler. (3) Hotels (manual working bench). (4) Position of microscopy station. (5) Position of liquid dispenser. (6) Automated work bench. Coordinates shown here are consistent with the remainder of the manuscript. See also Figs S1 and S2.

The Pelican is composed of a sterile automation enclosure that abuts a sterile manual enclosure on one side and an incubator on another. The automation enclosure contains a set of devices that may physically communicate via a four-axis gantry robot within a customised housing support. The manual enclosure is a cell culture hood, adapted for restricted communication of material with the automation enclosure. The automation enclosure currently includes a de-lidder, eight-fold and 96fold parallel dispenser, three-axis fourfold liquid handling robot (pipettor) with disposable tips, confocal microscope, and camera. The assembly (Fig. 7a,b), all of the components are described in the Supplementary Experimental Procedures.

### Cell culture

All work with human iPSCs and thereof derived cells has been approved by the Ethics Review Panel (ERP) of the University Luxembourg as well as by the Luxembourgish Comité National d’Ethique de Recherche (CNER). The CNER reference number is 201305/04. All experiments were performed in accordance with relevant guidelines and regulations. We confirm that written informed consent was obtained, by our cell line suppliers, for the establishment of stem cell lines, from all donors.

#### Human neuroepithelial stem cell culture

In order to demonstrate the capability of the automated system to maintain and monitor many types of cells including PD specific cell lines, we used a human neuroepithelial stem cell line from a healthy donor (hNESC K7) and a human neuroepithelial stem cell line derived from a patient carrying the Parkinson’s disease related mutation p.I368N in PINK1 (40066C5N). These cells were maintained and differentiated into midbrain-specific dopaminergic neurons within an OrganoPlate, by automating an existing macroscopic cell culture protocol^20^, that we previously adapted for microfluidic cell culture^35^. In brief, to culture hNESCs in an OrganoPlate, they were harvested from wells of a 6 well plate. The harvested hNESCs were then re-suspended on Matrigel (catalogue number 354277, lot number 3318549, Discovery Labware, Inc., Two Oak Park, Bedford, MA, USA). 0.7 μL of this Matrigel-cell mix was loaded in assigned chips of the OrganoPlate at a density of 0.03 million cells/μL. After seeding the cells, the plate was loaded into position B3 in the hotel of the Pelican. Afterwards, the plate was moved by the robotic arm to the storage incubator, at 37 °C and 5% CO2.

#### Dopaminergic neuronal differentiation

The culture medium preparation “N2B27 medium” consisted of mixed equal amounts of Neurobasal medium (invitrogen/life technologies) and DMEM/F12 medium (invitrogen/life technologies) supplemented with 1% penicillin/streptomycin (life technologies), 2 mM L-glutamine (life technologies), 0.5 X B27 supplement without Vitamin A (life technologies) and 0.5 X N2 supplement (life technologies). The medium to maintain the hNESC in culture “maintenance medium” consisted of N2B27 medium with 0.5 μM PMA (Enzo life sciences), 3 μM CHIR (Axon Medchem) and 150 μM Ascorbic Acid (Sigma Aldrich). The differentiation medium formulation to induce the differentiation of hNESCs towards midbrain dopaminergic neurons “differentiation medium with PMA” consisted of N2B27 medium with 200 μM ascorbic acid, 0.01 ng/μL BDNF (Peprotech), 0.01 ng/μL GDNF (Peprotech), 0.001 ng/μL TGFβ3 (Peprotech), 2.5 μM dbcAMP (Sigma Aldrich) and 1 μM PMA. The function of PMA in this medium preparation was to stimulate the sonic hedghog (SHH) pathway in the cultured hNESCs. Differentiation medium with PMA was changed, every 2 days during the first 6 days of culture in the differentiation process. For the maturation of differentiated neurons, PMA was no longer added to the differentiation medium “differentiation medium without PMA” from day 7 onwards, which was changed every 2 days during 3 weeks. To monitor cellular morphology during differentiation, bright field images were acquired automatically in the Pelican using the microscopy station.

#### Calcium imaging assay

A calcium imaging assay was done on 15 representative chips of the OrganoPlate at day 24 of differentiation. At room temperature, 50 μL of 5 μM cell permeant Fluo-4 AM (Life technologies) in neurobasal medium (Invitrogen/Life technologies) was manually added to the medium inlet well and 20 _μ_L to the medium outlet well of selected chips of the OrganoPlate. Then, the plate was incubated for 30 min at 37 °C and 5% CO2. The plate was then placed in an onstage incubator within the microscope. Calcium time-series of spontaneously firing hNESC-derived neurons were then automatically acquired. Images were sampled at a rate of 1 Hz for approximately 5 min, stored as image stacks and analysed using custom Matlab (version 2016b; MathWorks Inc.) scripts. Regions of interest corresponding to individual cells were automatically segmented with an established technique^37^ and fluorescence traces were generated for each segmented cell and presented as relative changes in fluorescence intensity ∆*F/F*.

#### Immunofluorescence staining assay

Immunostaining for the dopaminergic neuronal markers class 3 beta tubulin (TUB_β_III) and tyrosine hydroxylase (TH), the penultimate enzyme in the biosynthesis of dopamine^20,49,50^, was performed on representative chips at day 24 of differentiation. Differentiated cells were fixed with 4% paraformaldehyde (PFA) in 1 × phosphate-buffered saline (PBS) for 15 min, by manually adding 70 μL in medium well inlet and 30 μL in medium well outlet followed by permeabilisation with 0.05% Triton-X 100 in 1 × PBS (3 min on ice), and blocking with 10% fetal calf serum (FCS) in 1 × PBS (1 h). After washing with 1 × PBS, the primary antibodies mouse anti-TUBβIII (1:2000, Covance) and rabbit anti-TH (1:2000, Santa cruz biotechnology), were incubated for 90 min at room temperature. After washing with 1 × PBS, the secondary antibodies Alexa Fluor 488 Goat Anti-Mouse and Alexa Fluor 568 Goat Anti-Rabbit together with a stain DNA (Hoechst 33342, Invitrogen), were incubated for 2 hours at room temperature. After washing with 1 × PBS and water, confocal images of representative culture chambers were acquired using a confocal microscope (Zeiss LSM 710).

### Automating the differentiation of human neuroepithelial stem cell into dopaminergic neurons

LACS Config Editor was used to develop automated pipelines for the differentiation of hNESCs into dopaminergic neurons and time-lapse imaging microscopy. The automated pipelines were drafted according to the SiLA communication protocol and command format as previously described^9,42,51^. In brief, SiLA uses a Simple Object Access Protocol (SOAP) and a Web Service Description Language (WSDL) documentation, both of which are based on XML. A full library of commands for each device is downloaded once and stored in LACS as a configuration document of the Pelican. LACS Config Editor was used to incorporate the automated pipelines in this configuration file used herein to automate the differentiation of hNESCs into dopaminergic neurons. On the workbench, gel-embedded hNESCs were manually loaded into the culture lanes of a 2-lane OrganoPlate as described above. Then, the plate was put inside the Pelican through Hotel B, and placed inside the storage incubator by the robotic arm. The dispenser was fitted with a 5 μL cassette from Biotek and used as a dispensing medium for the media change.

### Statistical Analysis

Three representative chips (n = 3) were selected to illustrate the results of this study, in which the statistical analyses were performed by determination of the mean value and the standard deviation of the proportion of dopaminergic neurons within the overall neuronal population.

## Electronic supplementary material

Supplementary Video 1

Supplementary Video 2

Supplementary Video 3

Supplementary Material

## Data Availability

All data used within this study are available.
