# Supplementary material for: Automated microfluidic cell culture of stem cell derived dopaminergic neurons
Source: Sci Rep. 2019 Feb 11;9:1796. doi: 10.1038/s41598-018-34828-3 (PMC6370836; doi:10.1038/s41598-018-34828-3)
Supplement: Supplementary file 4 — Supplementary Material [file 41598_2018_34828_MOESM4_ESM.pdf]

Automated microfluidic cell culture of stem cell derived dopaminergic neurons

Khalid I.W. Kane, Edinson Lucumi Moreno, Siham Hachi, Moriz Walter
Javier Jarazo, Thomas Hankemeier, Paul Vulto, Jens C. Schwamborn,
Martin Thoma, Ronan M.T. Fleming

Supplementary Material

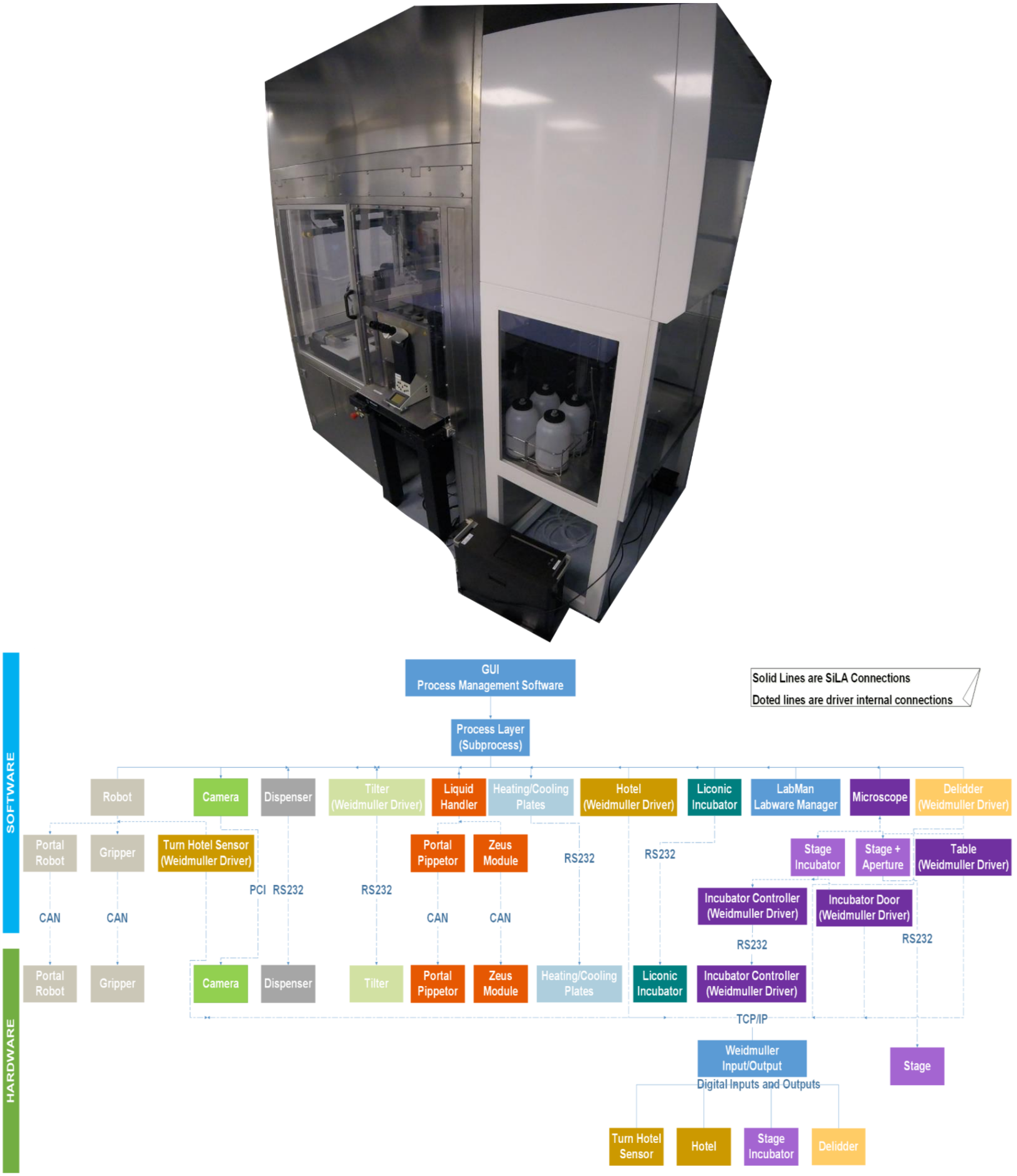

Figure S1: Pelican hardware and software control architecture. Standard on arrow = type of physical connection between a device and the computer. CAN = Controller Area Network. PCI = Peripheral Component Interconnect. RS232 = Recommend Standard number 232. TCP/IP = Transmission Control Protocol/Internet Protocol.

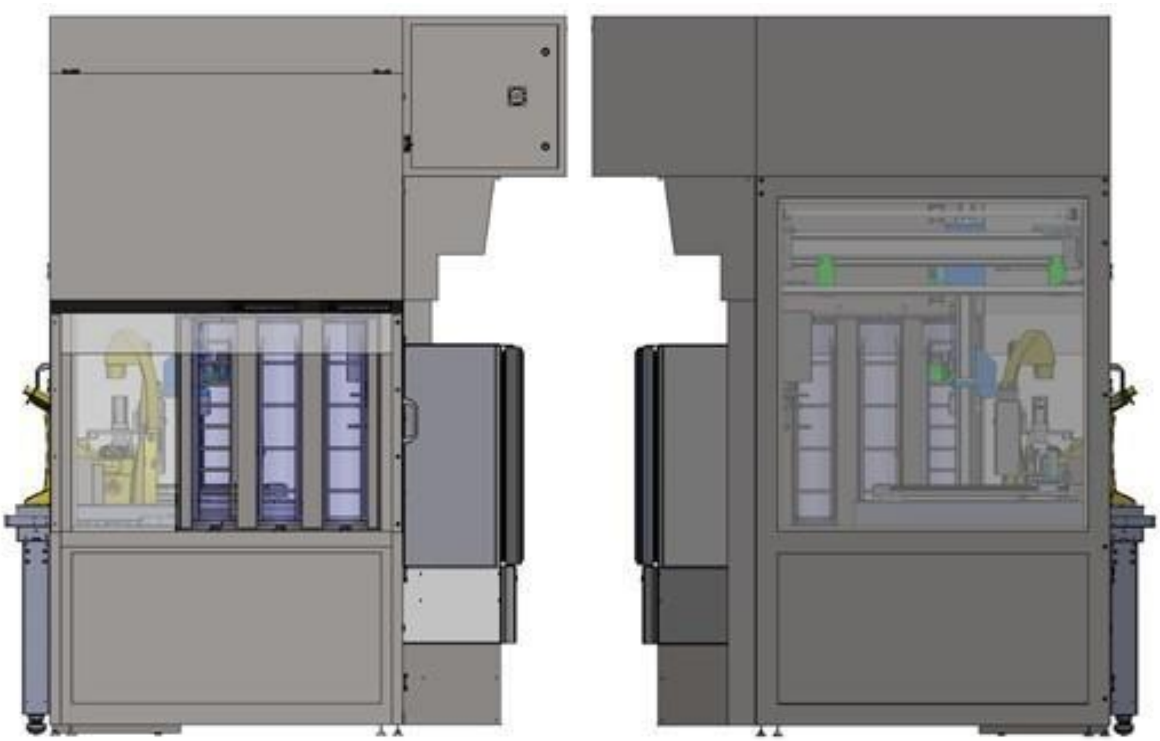

Figure S2: Left and right views of Pelican with housing illustrating the manual work bench and hotels (Left) that connect the Pelican to the outside world and the automated liquid handling station (Right).

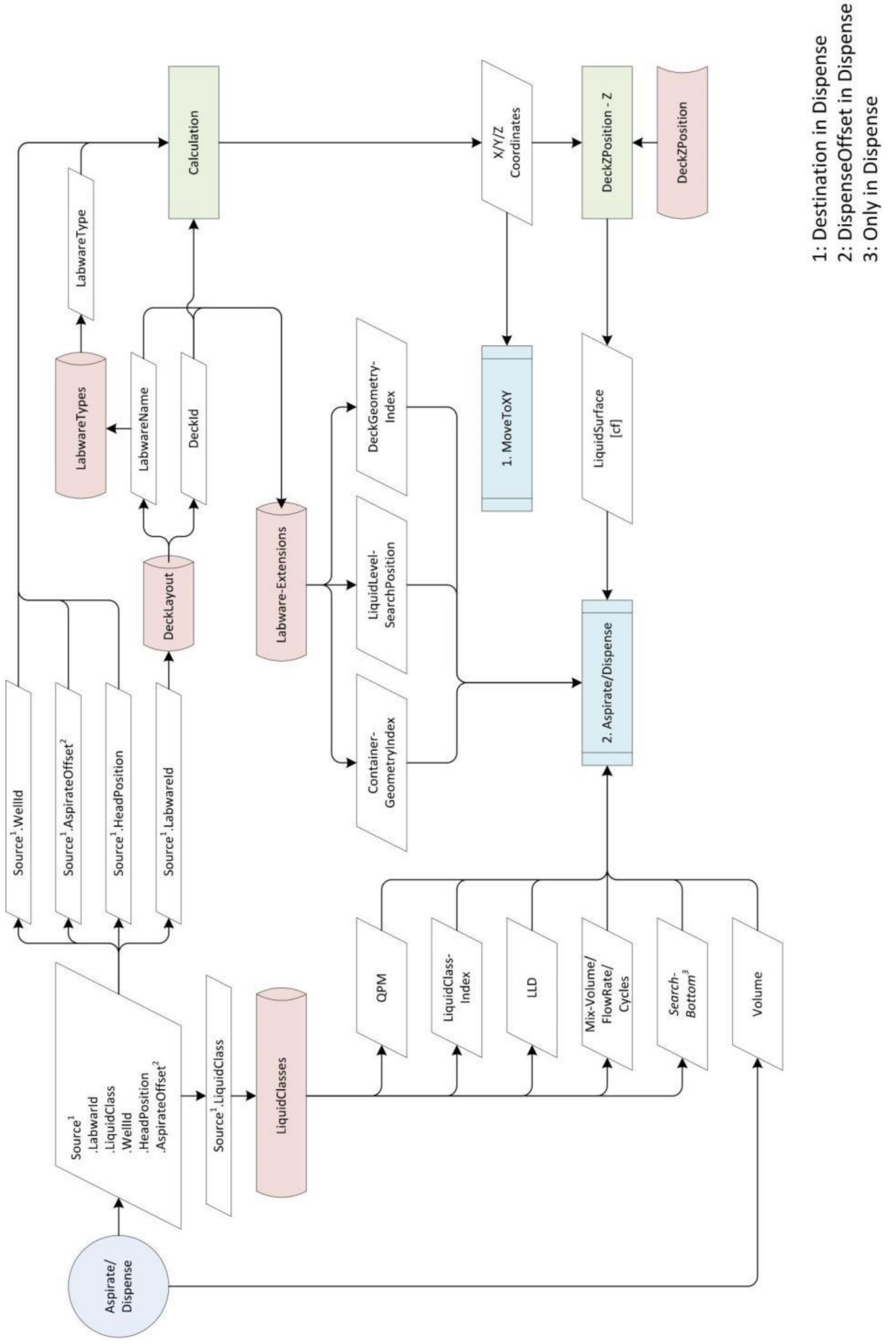

Figure S3: Liquid handler data model for pipetting protocols. Blue (physical action of pipette), red (passive action of pipette: reading from liquid-handler database) and green (passive action of pipette: determining well geometry). QPM=Qualitative Pipette Monitoring. LLD= Liquid Level Detection. The automated pipetting follows this order: pickup a disposable tip, detect the liquid level, aspirate liquid, dispense the liquid into the destination well and finally discard the tip.

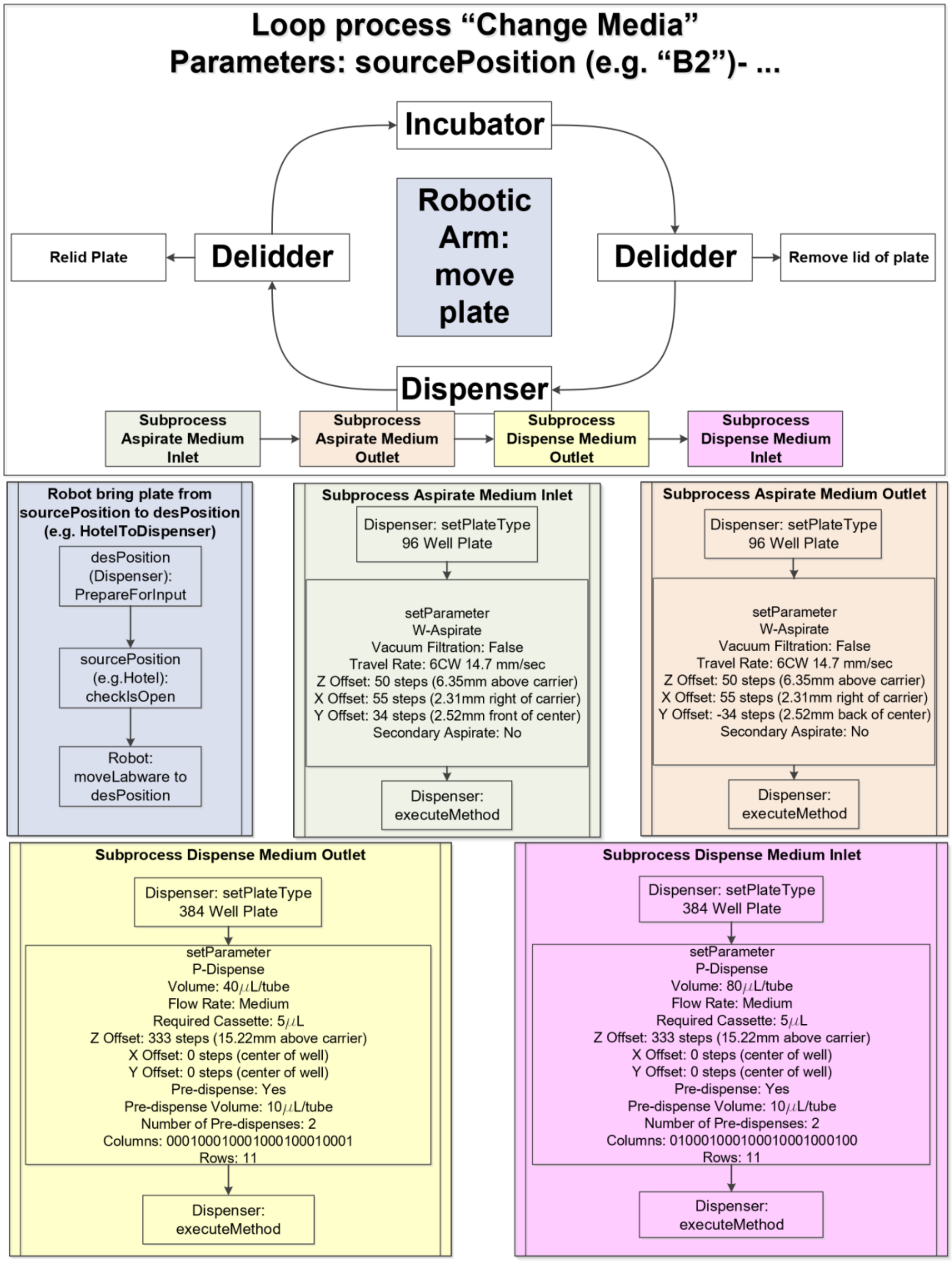

Figure S4: **Automated media change pipeline.** W = Washer, P = Peristaltic, 0 = Method not applied to column, 1 = Method applied to column, -1 = search position. Curved connection = Plate movement. Straight connection = Device specific method.

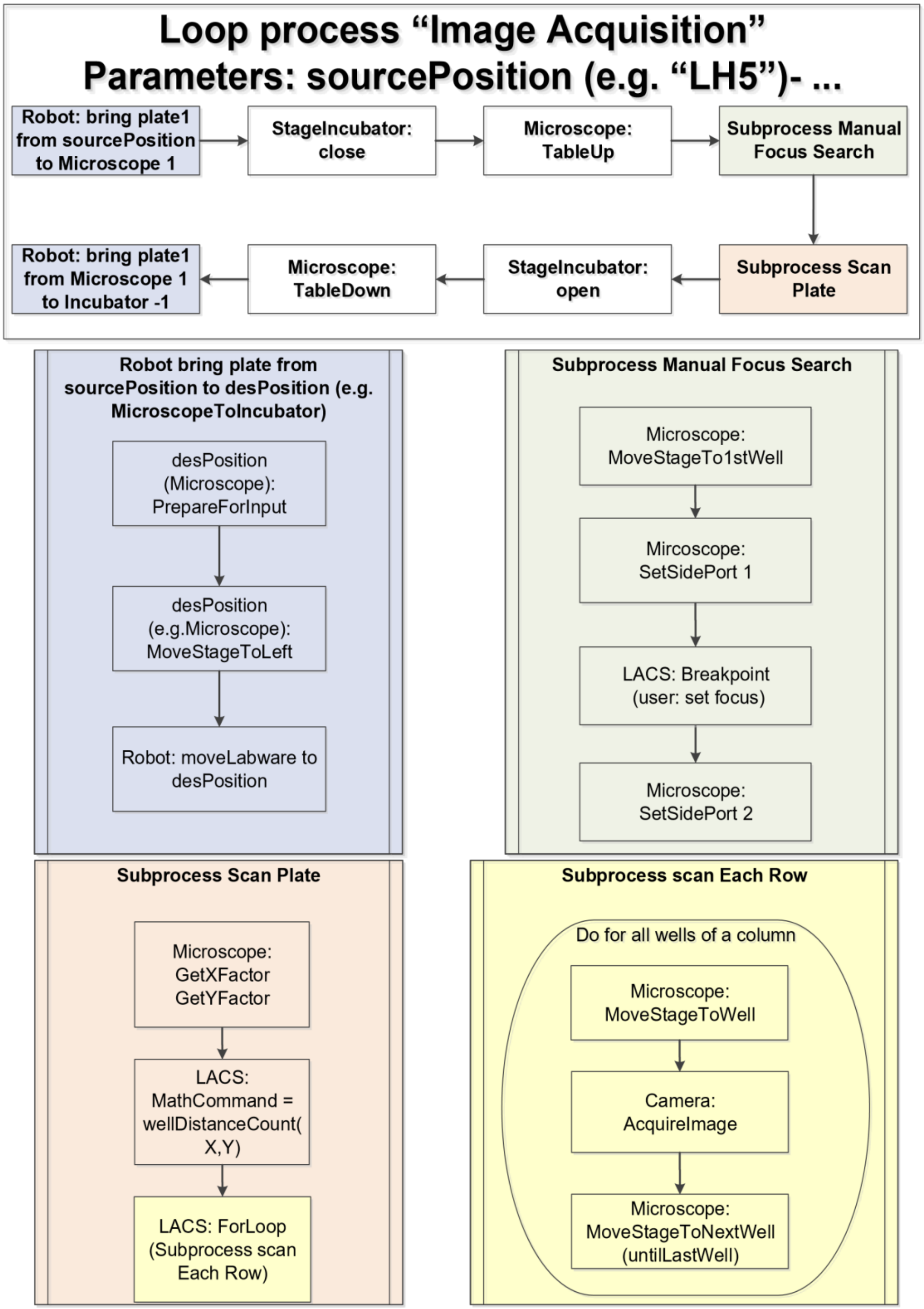

Figure S5: **Automated image acquisition pipeline.** Abbreviations as in Figure S4.

Supplementary Experimental Procedures

Hardware

Housing The sterile housing of the Pelican (Figure 6B) consists of a stainless steel frame support. The active components of the system, such as the dispenser, pipettor and de-lidder can be flexibly arranged on a perforated stainless steel work surface in any configuration compatible with accessibility via the four-axis gantry robot (Figure 6). This work surface is laterally bordered by an enclosure that guards against manual collision with any robotic moving parts. Large Plexiglas 360º viewing windows enable observation of enclosed processes. The passive components of the Pelican such as the control system, switch cabinets and a variable collection of waste containers (Figure 6B), are housed beneath the work surface.

Robot and grippers A customised four-axis gantry robot (Festo GmbH & Co. KG, Karlsruhe, Germany) was incorporated and mounted at the top of the stainless steel frame support inside the housing. The robot is used to transport any Society for Biomolecular Screening (SBS) format plate (ANSI SLAS 4-2004) and consumables by way of a gripper. The plates were processed at the individual stations, then transported by the robotic arm. The robot and the gripper perform all handling and transport functions of the platform that are not already covered by an individual device. Customised gripper jaws (Fraunhofer IPA, Fraunhofer Society, Stuttgart, Germany) were implemented in order to cover the multitude of plate formats with one gripper. Plates and carriers for consumables can be gripped and stacked by this robot.

Liquid-handler (Supplementary Video 3). A pipettor transfers various reagents in and out of almost any plate types. Every pipetting step was executed via an air displacement pipetting arm (LiHa-Arm 4x, Hamilton Bonaduz AG, Bonaduz, Switzerland) with disposable tips similar to hand pipettes. Four pipetting units (Z-Excursion Universal Sampler, Hamilton Bonaduz AG, Bonaduz, Switzerland) called ZEUS modules were mounted in a customised X-Y portal (Festo GmbH & Co. KG, Karlsruhe, Germany). Even though the liquid handling robot can move in the X-Y directions, it cannot transport plates. The transport of plates is realised via the four-axis gantry robot. The liquidhandler is equipped with both a capacitive and a pressure liquid level detection system. The capacitive technology is used to detect the liquid level of conductive liquids, while the pressure based technology is for both conductive and non-conductive liquids. Each ZEUS module has an anti-droplet control technology preventing cross contamination from a leaking tip. The pipettor is used to execute precise (pipetting resolution of $\pm 0.1\mu L$) liquid handling steps. It is able to handle volumes from $1\mu L$ to $1000\mu L$ depending on the available tip sizes in the plant. Depending on volume and the liquid used, contact and non-contact dispensing is possible. The liquid handlers is equipped with 5 positions to hold racks of 96 tips each, a tilting module and 4 heating and cooling stations, the latter 2 are described below. A graphical user interface that follows a specific structure (see Figure S3) was implemented to write all pipetting protocols.

Tilter A Multiflex Tilt Module (Kust, Hamilton Bonaduz AG, Bonaduz, Switzerland) was installed on the liquid handling deck, and is used to tilt SBS plates between a horizontal and a 10° degree incline to completely remove the liquid out of big wells.

Heating and cooling positions Four CPAC Ultraflat & CPAC Microplate Inheco thermoblocks (INHECO Industrial Heating & Cooling GmbH, Planegg, Germany) units, each fitted with a Flat Bottom Adapter (7900016, INHECO Industrial Heating & Cooling GmbH) with positioning frame were installed on the deck of the pipettor as heating and cooling stations. They are used to control the temperature of four SBS plates (4°C-80°C) during time consuming liquid handling protocols. The thermoblocks are controlled by a Multi TEC Control Unit (8900030, INHECO Industrial Heating & Cooling GmbH).

Liquid dispenser A dispenser (EL406, BioTek Instruments Inc, Bad Friedrichshall, Germany) is used to transfer various reagents in and out of SBS plates. It is less precise than the liquid-handler. The liquid dispenser has a dispensing resolution of 2% to 11% depending on the protocol. However, it is much faster than the liquid-handler as it can handle up to 96 wells per step compared to 4 wells per step. For washing steps 96 wells can be filled and emptied simultaneously or with the dispensing unit 8 wells can be filled parallel.

Microscopy A microscope (DMI6000B, Leica AG, Wetzlar, Germany), fitted with an automated onstage stage incubator in an automated stage (SCAN IM 597382, Marzhauser Wetzlar GmbH & Co. KG, Wetzlar, Germany), and a camera (Neo sCMOS 5.5, Andor Technology Ltd, Belfast, Northern Ireland), is used to record images from culture plates. The microscopy assays can be run for a long period of time without perturbing the optimal cell culture conditions because of the presence of the onstage incubator that regulates the CO_2 and the temperature levels.

Onstage incubator A small incubator housing (H301-K-Frame, Okolab SRL, Naples, Italy) was installed on the stage of the microscope. This incubator was used to store plates in SBS format under regulated conditions. The temperature and CO_2 of the onstage incubator is controlled during microscopic examination by a temperature controller (H301-T-UNIT-BL-PLUS Boldline, Okolab SRL, Naples, Italy) and a CO_2 controller (0506.000, Pecon GmbH, Erbach, Germany) respectively.

Storage Incubator An incubator (STX-44, LiCONiC AG, Mauren, Principality of Liechtenstein) is used to store plates in SBS format under regulated conditions in terms of temperature, CO_2 and humidity. A varying number of plates, of varying height, may be stored depending on the format of the racks within the incubator. Two standard Liconic stackers, each with a pitch of 23mm suitable for plates with a maximum height of 17mm, were mounted inside the incubator. Each stacker can store up to 22 plates.

de-lidder Two de-lidders are used to remove the lids of up to two SBS plates located in the process at any given time. The lids are kept by the de-lidder while the plates are being processed. Another function of this component is to place the lids back on after the plates were processed. It has an internal pressure-based check that verifies the correct handling of the lids for removal and closure.

Hotel The hotels are used as a secure transfer station between the outside world and inside the housing. All consumables and culture plates are fed in and discharged via the hotels. There are 3 hotels (A, B, and C). Hotel A and hotel B are intended for the loading and discharge of pipette tip racks. Pipette tips may be incorporated on four racks each with 96 tips. Plates in SBS format may be fed in and discharged in all hotels.

Waste Handling A waste position on the liquid handling deck is available for tip disposal while running a liquid handling process. Pipette tips exit via a downpipe under the work surface where they are dropped into a collection container, which can easily be pulled forward on a rail between the switch cabinets (Figure 6B). Media waste is collected in SBS plates, and removed through the hotels.

Environmental control

Sterility All the components of the Pelican were contained within an enclosure housing as described above. Air flows at a rate of pressure 161 Pa and wind speed of 0.45 m/s through a filter at the top of the automation enclosure, with a continuous laminar flow down through the perforated work surface, except where devices deviate the air flow. This air flow is designed to maintain sterility (biosafety level 1) of the automation enclosure.

Temperature The temperature levels were controlled on most positions of the Pelican. The temperature was regulated through the four heating and cooling positions (4°C-80°C) while changing the media on the liquidhandler deck. It was also controlled on the stage incubator (25°C-45°C), while acquiring images, and the incubator is always kept at $37°C$ at all times.

Carbon dioxide The Pelican was fed with pure carbon dioxide (99.99%) at a pressure of $1.5 bar$. The carbon dioxide levels inside the onstage incubator was regulated (0-7%) and the storage incubator was kept at 5% CO_2at all times.

Humidity While plates were stored inside the storage incubator, the relative humidity was kept at 95%.

Practicability A user manual details the procedure for operation of the Pelican Cell culture Observatory (available upon request). This document detailed not only how to operate the system, but also how to extend the current setup to include more devices, and how to integrate the said devices in terms of hardware and software development.
